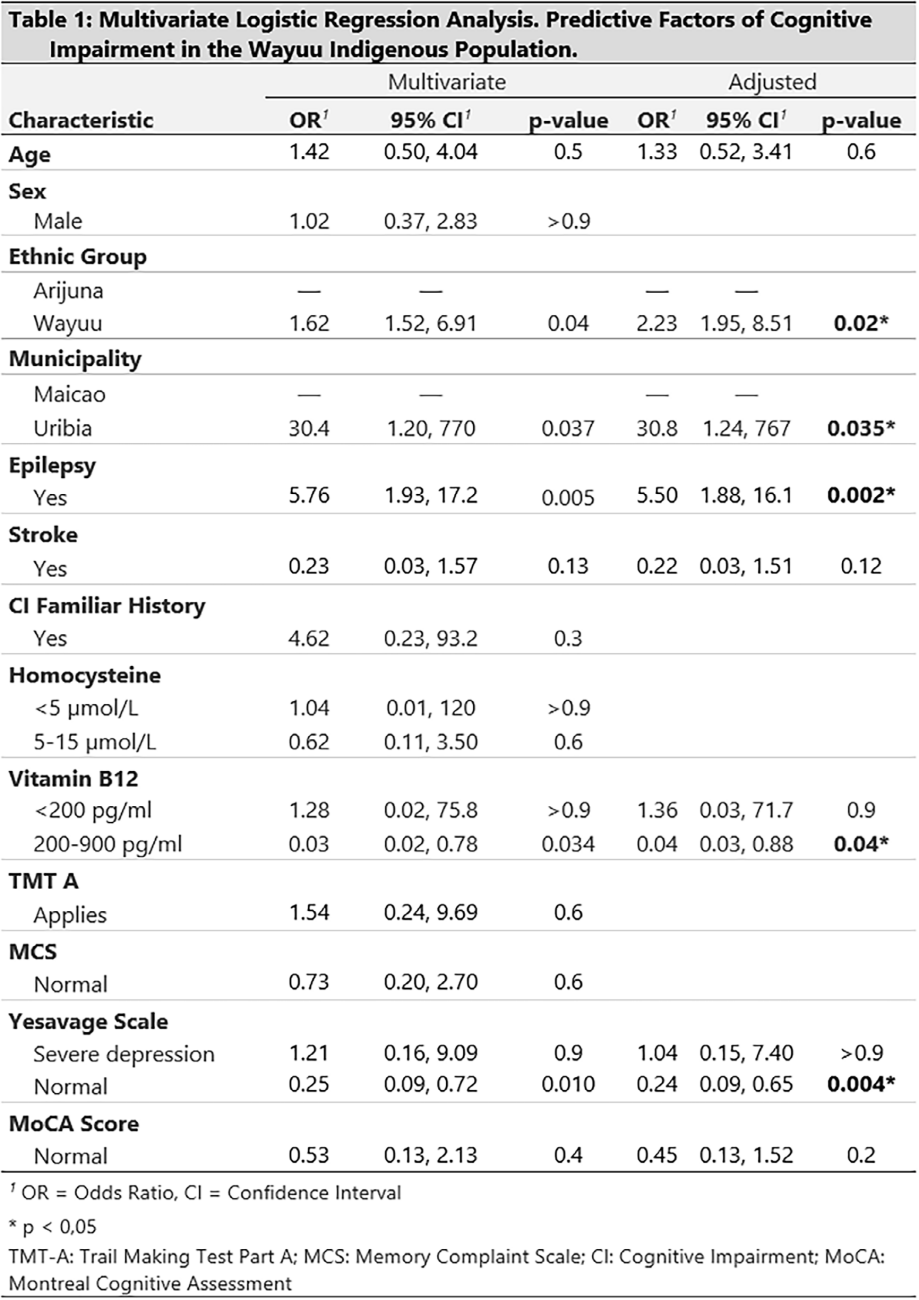# Evaluating Predictors of Cognitive Impairment: Insights from a Cross‐Sectional Analysis in the Wayuu Indigenous Population

**DOI:** 10.1002/alz.084766

**Published:** 2025-01-09

**Authors:** Jose Hernandez Preciado, Wanda Torres, Alex Dominguez Vargas, Yesenia Pianneta, Mauricio Medina, Marybel Sinisterra, José Vargas

**Affiliations:** ^1^ Universidad de Maimónides, Buenos Aires Argentina; ^2^ Universidad Simón Bolívar, Barranquilla Colombia; ^3^ Universidad del Norte, Puerto Colombia Colombia; ^4^ Universidad de Caldas, Manizales Colombia

## Abstract

**Background:**

Cognitive impairment (CI) encompasses a wide range of symptoms and signs associated with a progressive decline in cognitive functions, which adversely affect the quality of life and autonomy of patients. Indigenous communities have been found to have a higher prevalence of CI and dementia. However, there is limited knowledge regarding the prevalence of CI in ethnically diverse populations, such as the Wayuu indigenous community. The aim of this study was to identify predictive factors of cognitive impairment in the Wayuu indigenous population.

**Methods:**

A cross‐sectional study. We included 100 Wayuu indigenous and 100 non‐indigenous individuals from the northern coast of the Colombian Caribbean. Cognitive function was assessed using the Prolonged Latency Test through electroencephalography, and it was categorized as follows: normal, low beta activity (mild CI), frontal slowing (moderate CI), and alterations in alpha activity (severe CI). Sociodemographic, clinical, cognitive, and functionality variables were evaluated. Multivariate logistic regression analysis was conducted to evaluate predictive factors associated with CI.

**Results:**

A higher prevalence of moderate and severe CI was observed in the Wayuu indigenous population compared to non‐indigenous individuals (90% vs. 47% and 5% vs. 4%, p<0.05, respectively). Wayuu ethnicity (OR: 2.23, 95%CI: 1.95‐8.51, p = 0.02), epilepsy (OR: 5.5, 95%CI: 1.88–16.1, p = 0.006), and residing in the town of Uribia (OR: 30, 95%CI: 1.24‐767, p = 0.03) were identified as risk factors for CI. Normal levels of vitamin B12 (200‐900 pg/ml) (OR: 0.04, 95%CI: 0.02‐0.96, p = 0.04) and normal results in the Yesavage test (OR: 0.24, 95% CI: 0.09–0.65, p = 0.004) were found to be protective against CI.

**Conclusions:**

This study highlights the need to address epilepsy, depression, and vitamin B12 deficiency in Wayuu indigenous communities to design cognitive health intervention strategies.